# Is research working for you? validating a tool to examine the capacity of health organizations to use research

**DOI:** 10.1186/1748-5908-4-46

**Published:** 2009-07-23

**Authors:** Anita Kothari, Nancy Edwards, Nadia Hamel, Maria Judd

**Affiliations:** 1 University of Western Ontario, Arthur and Sonia Labatt Health Sciences Building, Room 222 London, Ontario, N6A 5B9, Canada; 2 University of Ottawa, 451 Smyth Road, Ottawa, Ontario, K1H 8M5, Canada; 3 University of Ottawa, 1 Stewart Street, Ottawa, Ontario, K1N 6N5, Canada; 4 Canadian Health Services Research Foundation, 1565 Carling Avenue, Suite 700, Ottawa, K1Z 8R1, Ontario

## Abstract

**Background:**

'Is research working for you? A self-assessment tool and discussion guide for health services management and policy organizations', developed by the Canadian Health Services Research Foundation, is a tool that can help organizations understand their capacity to acquire, assess, adapt, and apply research. Objectives were to: determine whether the tool demonstrated response variability; describe how the tool differentiated between organizations that were known to be lower-end or higher-end research users; and describe the potential usability of the tool.

**Methods:**

Thirty-two focus groups were conducted among four sectors of Canadian health organizations. In the first hour of the focus group, participants individually completed the tool and then derived a group consensus ranking on items. In the second hour, the facilitator asked about overall impressions of the tool, to identify insights that emerged during the review of items on the tool and to elicit comments on research utilization. Discussion data were analyzed qualitatively, and individual and consensus item scores were analyzed using descriptive and non-parametric statistics.

**Results:**

The tool demonstrated good usability and strong response variability. Differences between higher-end and lower-end research use organizations on scores suggested that this tool has adequate discriminant validity. The group discussion based on the tool was the more useful aspect of the exercise, rather than the actual score assigned.

**Conclusion:**

The tool can serve as a catalyst for an important discussion about research use at the organizational level; such a discussion, in and of itself, demonstrates potential as an intervention to encourage processes and supports for research translation.

## Background

Many factors have contributed to the increased interest in using health services research for administrative, clinical, and policy decisions. Growing expectations of accountability for public sector spending, the complexity of health systems tackling emergent health issues and demographic shifts, and the evolution of knowledge synthesis techniques all underlie the push for evidence-informed decision-making. Health system decision-makers around the world are committing to evidence-informed decision-making as sound and responsible practice [1-5].

Most of the focus of evidence-informed decision-making has been on clinical practice and evidence-based medicine. Other decision-makers – health system executives, managers, and politicians – make decisions that are every bit as critical as those of the practitioner. Senior health system administrators and managers make decisions ranging from day-to-day operations to longer-term strategic planning priorities. Politicians are responsible for defining priorities and the boundaries of programs and policies, with implications for on-the-ground health services delivery, financing, and program development. We submit that decision-makers at different system levels synergistically contribute to an organizational culture that may be more or less welcoming of research evidence use. In turn, an organization's structures and processes contribute to the ability of individuals to carry out research-informed activities.

An organization's capacity to facilitate the application of evidence is complex, and not well understood. There is substantial literature on decision support tools (*e.g*., clinical practice guidelines, electronic reminder systems, simulation models) [6-8]. Many of these tools may help an individual determine how well they are able to access, use, and understand research evidence, but there are few tools that have been developed for use at the organizational level. To accomplish this, we need to understand the processes and routines used at the organizational level.

The Canadian Health Services Research Foundation has conceptualized 'organizational research use' as an iterative process that involves acquiring, assessing, adapting, and applying research evidence to inform health system decisions. To improve evidence-informed decision-making at this broader level requires a better understanding of the processes and routines related to the use of health services research in an organization. In other words, the commitment to evidence-informed decision-making first requires taking stock of facilitators and challenges facing those who could potentially use evidence to make decisions. By taking stock, concrete ideas can be developed to support the acquisition, assessment, adaptation, and application of research findings. Thus, the foundation's vision of an organization that uses research is one that invests in people, processes, and structures to increase their capacity to use research.

The purpose of this paper is to describe the response variability, differentiability, and usability of a self-assessment tool for organizations to evaluate their ability to use research findings. The Canadian Health Services Research Foundation originally developed the tool. The mission of the foundation is to support evidence-informed decision-making in the organization, management, and delivery of health services through funding research, building capacity, and transferring knowledge.

### Organizations and the use of research

The implementation of evidence-informed decision-making in health care organizations is unlikely to follow the clinical model of evidence-based medicine. Individuals cannot adopt or implement research findings on their own; they require organizational support and resources. To illustrate, in one study, the characteristics of research per se did not fully explain the uptake of research findings whereas users' adoption of research, users' acquisition efforts, and users' organizational contexts were found to be good predictors of the uptake of research by government officials in Canada [9]. Further, empirical work in the field of organization and management clearly shows that successful individual adoption is only one component of the assimilation of innovations in healthcare organizations [10]. Yet, studies of individuals as adopters of research have generally not addressed the potential role of organizational elements that could be harnessed to influence the adoption process [11].

Recent frameworks related to the implementation of research or innovations are beginning to consider those organizational elements that act as barriers or facilitators to the uptake and use of research by individuals [12-14]. Authors have discussed the importance of such things as organizational structural features, culture and beliefs, leadership style, and resources (described in more detail below). Of note is that some of these frameworks collapse the distinction among the different types of decision-makers who might be supported in the use of research; we also took this generic approach when we evaluated the 'Is research working for you' tool in various settings.

Studies have demonstrated associations among organizational variables and the diffusion of innovations (*e.g*., an innovation might be a clinical practice guideline reflecting new research). Systematic reviews have identified some organizational features that are implicated in the successful assimilation of an innovation. Structural determinants, such as large organizational size and decentralized decision-making processes, were found to be significantly associated with the adoption of innovations [15,16]. Organizational complexity, indicated by specialization, professionalism, and functional differentiation, were also associated with innovation diffusion [17]. Resources and organizational slack are needed to introduce and support new innovations, as well as to provide monetary reimbursement for those professionals or their organizations that incorporate innovations into their routines [15,18].

There are also two non-structural determinants that have an impact on what is called organizational innovativeness: absorptive capacity and receptive context for change [15]. The organization's capacity to absorb innovation is its ability to acquire, assimilate, transform, and exploit new knowledge; to link it with its own prior related knowledge; and to facilitate organizational change [19]. Thus, an organization that supports and encourages innovation, data collection and analysis, and critical appraisal skills among its members will be more likely to use and apply research evidence [20]. The receptive context for change refers to the organization's ability to assimilate innovations by providing strong leadership, clear strategic vision, and possibility for experimentation.

While it is difficult to draw definitive conclusions from primary innovation studies due to their methodological weaknesses [18], it does seem to be the case that the user's system or the organizational context seems to be one of the major determinants that affects the assessment, interpretation, and utilization of research. These findings imply the need to commit organizational resources to ensure successful adoption of research findings for effective decision-making by the individual within the organization [21,22]. Resources need to be accompanied by strategies that will go beyond the individual and consider the collective for a culture of evidence-informed decision-making. One promising view of how organizations should effectively learn and manage knowledge, 'learning organizations' [23], may be helpful for enabling the use of research in decision-making. Learning organizations are characterised as organizations that stimulate continuous learning among staff through collaborative professional relationships across and beyond organizational levels. Moreover, individual goals are aligned with organizational goals, and staff is encouraged to participate in decision-making, which in turn promotes an interest in the future of the organization [23]. Another pertinent perspective is Nonaka's theory of collective knowledge creation [24]. Through 'fields of interactions', individuals exchange and convert explicit and tacit knowledge, thereby creating new collective (organizational) understandings. Both learning organizations and the theory of knowledge creation emphasize the need for on-going social interactions in order for knowledge to spread from the individual user to groups of users, which in turn can affect organizational structures and processes.

Decision-makers can increase their ability to identify and assess new knowledge generated from research activities and use that knowledge to enhance their organizational capabilities. A first step in this change process is to examine an organization's capacity to access, interpret, and absorb research findings.

### Development of the tool

The self-assessment tool 'Is research working for you? A self-assessment tool and discussion guide for health services management and policy organizations' was developed by the Canadian Health Services Research Foundation and colleagues in response to requests for assistance from Canadian health service delivery organizations in identifying their organization's strengths and weaknesses in evidence-informed decision-making. The tool was designed to help organizations examine and understand their capacity to gather, interpret, and use research evidence. Accordingly, in this paper, we are narrowly defining 'evidence' to mean scientific findings, from research studies, that can be found in the academic literature and in the unpublished literature (*e.g*., government reports).

Development of the tool involved an iterative process of brainstorming, literature reviews, focus groups, evaluations of use, and revisions. Development started in 1999 with the first version of the self-assessment tool that was informed by a review of the health literature on the major organizational capabilities for evidence-informed decision-making [25]. The result was a short, 'self-audit' questionnaire that focused on accessing, appraising, and applying research. In 2000, the questionnaire was revised based on review of the business literature that encompassed topics such as organizational behaviour and knowledge management [26]. As a result, the questionnaire's three A's (accessing, appraising, and applying) were supplemented with another A – adapting. Focus groups with representatives from regional health authorities, provincial ministries of health, and health services executives provided feedback on the strengths and weaknesses of the instrument. Adjustments to the wording of items on the tool were made based on focus group input. Further, revisions reflected the need to create a group response with representatives from across the levels of the organization because both literature reviews and focus groups clearly indicated that while evidence-informed decision-making was often portrayed as a discrete event, it is in fact a complex process involving many individuals.

The tool itself is organized into four general areas of assessment. Acquire: can your organization find and obtain the research findings it needs? Assess: can your organization assess research findings to ensure they are reliable, relevant, and applicable to you? Adapt: can your organization present the research to decision makers in a useful way? Apply: are there skills, structures, processes, and a culture in your organization to promote and use research findings in decision-making? Each of these areas contains a number of items. For example, under 'acquire', users are asked to determine if 'we have skilled staff for research.' Each item uses a five-point Likert scale (where a one means a low capacity or frequency of activity, while a five signifies something the organization is well-equipped to do or does often).

An earlier version of the tool was used for this study; the revised, current version of the tool can be obtained by sending a request to research.use@chsrf.ca. More information about the tool is available at .

## Methods

### Objectives and design

The research objectives were to: determine whether the tool demonstrated response variability; describe how the tool differentiated between organizations that were known to be, *a priori*, lower-end or higher-end research users; and describe the potential usability of the tool within selected organizations in four health sectors. A mixed methods study design was used. Focus groups provided a rich source of qualitative data, while participants' responses to the tool yielded quantitative data. The study received ethics approval from the Health Sciences and Science Research Ethics board at the University of Ottawa.

### Study sample

Focus groups were conducted among four sectors of Canadian health organizations: selected branches of federal government, long-term care organizations, non-governmental organizations, and community-based organizations. Key advisors actively involved in each of the sectors identified organizations that were expected to be higher-end versus lower-end research users. Common descriptors of higher-end research users included those organizations with a medium- to long-term history of active participation in internally and externally funded research projects, and/or formal affiliations with a university and/or academics, and/or a history of presenting research and/or attending annual conferences. With respect to public health (as part of community-based organizations), university-affiliated health units in Ontario were categorized as higher-end research users and all other health units were categorized as lower-end research users.

The original aim was to recruit 40 organizations; ten from each of the four sectors. Our sampling frame for the community sector included 59 organizations; for the long-term care sector included 83 organizations; for the non-governmental organization (NGO) sector included 26 organizations; and for the government sector included 20 government departments/branches. Not all organizations were invited to participate: once it became clear that organizations in a sector were interested and that we were approaching or had approached our sample size goal, we stopped inviting new organizations. To recruit participants, an e-mail was sent to the contact person in a randomly selected organization within each sector. Through the contact person, each organization identified a small group of individuals (four to six) to represent the organization/branch's interests in research. They were asked to participate in a two-hour focus group on-site. A pre-determined leader from their group explained the procedures, and managed the first hour of the focus group. Participants were asked to work through the tool as if at a regular organizational meeting. They individually completed the tool (sometimes in advance of the meeting) and then they discussed the items and their rankings, and in most cases derived a group consensus ranking on items. The research team facilitator was present for the first hour of the focus group but did not contribute unless clarification about the procedures was required. In the second hour, the research team facilitator posed questions, asking group members to discuss overall impressions of the tool, identify insights that emerged during the review of items on the tool, and comment on areas of research utilization and capacity that may not have been addressed. Organizations were provided with a $250 incentive to offset the costs of staff participation.

When feasible, a facilitator and note-taker went to the participant site (n = 18). In some cases the focus group was conducted via teleconference (n = 14). Facilitators and note-takers produced a debriefing note after each session. All sessions were tape recorded and transcribed with the consent of participants. Respondents were asked to return copies of their completed tools to the research team. They were given these instructions either at the end of the focus group session or several weeks following the focus group.

### Data analysis

#### Qualitative analysis

A coding scheme was developed using two focus group transcripts by two independent investigators. All transcripts were subsequently coded using the predetermined coding scheme [27]. Categories and subcategories were thematically analyzed for emerging trends and patterns, with the assistance of N6 (NUD*IST) qualitative research software. Qualitative results are based on 32 transcripts.

#### Quantitative analysis

This was conducted using SPSS, statistical software, to compare the numerical ratings of items that were written on the tools and discussed during the focus groups. Information on two ratings was extracted. First, the individual ratings noted on the tool in advance of the focus group discussions were extracted. The returned tools (and in some instances, when the individual forms were not returned to us, the transcript) provided a record of these individual ratings. Second, the consensus ratings for each item on the tool were identified from either a written record of the consensus scores or the transcript.

Of the 32 focus groups, two groups (total of six participants) deliberately received a version of the tool that did not include the rating scale (*i.e*., only qualitative data available). Further the consensus scores of those who participated from the government sector were excluded from bivariate analysis due to small numbers of participants (six) and groups (two) for this sector. Thus, quantitative results for individuals are based on information from 30 focus groups, and results for consensus scores are based on information from 28 focus groups.

The variable for individual scores was coded as 'missing' for those individuals who did not return their tool or provide their ratings on their returned tools. The same consensus score for a questionnaire item was assigned for each member of that focus group. For some items, group members chose not to reach a consensus score. In these instances, the variable for consensus score was coded as 'missing'. In other instances, groups arrived at a consensus by assigning a score in-between ratings on the Likert scale. Thus, for example, some of the final consensus scores were 1.5 or 2.5. The consensus score was used for the focus group level of analysis. The range, mean, and standard deviation for each item on the individually completed and consensus-derived scores were computed to assess response patterns. Non-parametric statistics (Kruskal Wallis test) were used to compare the differences between higher- versus lower-end research use organizations for individual and consensus scores.

## Results

In terms of recruiting outcomes, of the 47 community organizations approached, 16 participated in the study; of the 83 long-term care organizations, 6 participated; of the 26 NGOs approached, eight participated; and of the 20 governmental departments/branches, two participated. During recruitment it was discovered that a Canadian Council on Health Services Accreditation process was occurring in the long-term care sector. Consequently, many long-term care organizations were unable to participate in the study. Other reasons for refusing to participate, that were common to all sectors, included lack of time, staff involvement in other research, and a perception that the project was not relevant to their organization (*e.g*., 'this doesn't apply to us'). A total of 142 individuals participated in the 32 focus groups. In total, 77 participants returned their individually completed tools to us, six participants had used a version of the tool without scales, and 59 did not return their tools or did not provide their ratings on their returned tools.

### 1. Response Variability of Tool

The tool data was complete (*i.e*., a response was noted for each item of the questionnaire) for 66 of the 77 participants who returned their tools to us. The items with the largest number of missing responses were for items 'evaluate the reliability of specific research by identifying related evidence and comparing methods and results' and 4.2C 'when staff develop or identify high quality and relevant research, decision-makers will usually give formal consideration to any resulting recommendations', each with eight missing responses, 10.4% of respondents. Individual participants used the full range of response options (one to four) for all items on the questionnaire. Average scores ranged from 1.9 (SD 0.79) to 3.21 (SD 0.6) for the items 'our organization's job description and performance incentives include enough focus on activities which encourage using research' and 'learning from peers, by formal and informal networks to exchange ideas, experiences, and best practices', respectively.

In comparison with individual responses, a truncated set of scoring options were often used by the group in arriving at consensus scores. For 15 of the 27 questionnaire items, consensus scores had a range of two (*i.e*., the final scores did not cover the full range of scoring options available). Consensus scores were missing for a number of reasons: the data were not extractable from transcripts in those cases where not recorded, the group chose not to give a consensus score to a particular item; or the group ran out of time and had no opportunity to discuss consensus scores for a particular item. In general, groups spent much more time discussing the first section of the questionnaire, and then quickly moved through the last two or three sections.

### 2. Differentiation between higher- and lower-end users of research

With the exception of two individual scores and four consensus scores, the average individual and/or consensus scores were higher for higher-end than lower-end research use organizations on every questionnaire item (See Additional File 1: Comparison of individual and consensus scores by higher versus lower end organizational research users for the original data). These differences were statistically significant for 13 of the 27 items individually rated, and for five of the 27 items rated by consensus. No consensus scores were significantly different between the two groups for sections three ('adapt research') or four ('apply research').

### 3. Potential usability

#### Access

Practically every single group described the lack of time they had in their workdays to access, read, and incorporate research into their tasks and decision-making (the general tone was not defensive but rather matter-of-fact). When probed, focus groups participants mentioned that while not everyone had the skills to access research (some participants were not sure they had the ability to even identify their research needs, or their researchable questions), there were some highly skilled people in an organization who were available to access research. Furthermore, there was an awareness of the research being available via internal databases and subscriptions. The impact on the budget was seen as important (the cost of maintaining electronic or print journal subscriptions), as noted by one participant: 'My budget for the whole hospital for acquisitions, including all my subscriptions and all my databases, is less than $50,000. These things just can't be bought on that sort of money' (FG 29). Another issue was trying to access those particular individuals or programs with the skills to help with retrieving and interpreting the research. Accomplishing this often required a formal request.

The participants also noted that the informal networks that they or their departments have with external, university-based researchers were very important. They saw this source as an effective way to find out about the literature in an area, about what the current position on an issue was, and what was seen as best practice.

#### Assess

Participants identified a general lack of skills around assessing the research. Those organizations that had individuals with the research transfer skills suggested that more mentoring needed to occur to help increase the skill base. Also, there was a suggestion to remind employees that using research is simply part of their job, or to make it an integral part of what is expected from the staff coming into the system (*i.e*., incorporated in a job description). One group discussed the fear that some may have in admitting that they lack the skill set required for using research, as described by one participant: 'I think we also have a fair number of people who are afraid to admit that they don't know how to look at and figure out if something is good science or not' (FG 29).

#### Adapt and apply

Focus group discussions revealed an even greater difficulty with adapting and applying the research. That is, there was issue with contextualizing the research findings, 'It is difficult [for] organizations at the grass roots to determine sometimes what stuff is relevant, which parts are relevant to what we are doing on a day-to-day basis' (FG 20). Participants were split about whether they were able to adapt research well. Some described organizational pockets that seemed to do a better job than others.

Research was not being adapted, however, on a regular basis. In many cases, the roadblock was having a stakeholder partner accept the evidence. Participants described how many factors played a role in decision-making, as illustrated in this participant comment: 'It's not that we doubt the evidence. It's that all those other factors, and I guess that's where...' (FG 21).

In terms of unique findings from the government sector, one participant suggested that senior bureaucrats do not value research and another said, 'policies are often out of sync with political dynamics' (FG 3). Consequently, participants did not feel that research was a high priority from the higher levels in the organization. Even though the opportunities were there – *e.g*. research forums – '...the culture forbids you from going because that's viewed as you can't be doing your job properly if you're not too busy' (FG 9). Various barriers were identified to using research in government. One of the prominent barriers was the idea that the lack of application might be due to the focus of the research available. It was thought that much of the current research did not address operational or practice issues, which would be of interest to government decision-making. The prevailing mood of the two focus groups in the government sector was that they did not find the tool useful.

What was unique about the long-term care sector was the perception that research use for decision-making might be occurring at the management level. In particular, participants talked about being 'handed down' best practices. On the other hand, there were occasions, participants noted, when management requested research from the lower levels. This was described as decision-makers wanting the 'right' information, the 'nitty-gritty'. Decision-makers wanted the research to help them put out fires. These groups identified a bit of trouble with the research terminology. The concept of adapting the research was the easiest for them to understand; many groups stated that they came to consensus faster at this point. As stated by one participant, '...it's not asking us about doing research or assessing research, it's can we adapt the format of research. And personally I feel more capable of doing that' (FG 15).

NGOs noted that the tool seemed to be geared to a more formal type of organization. Furthermore, the tool was focused on management and policy research, not the clinical practice research and the health policy economics issues that were of more central interest to them. Nevertheless, there was a strong feeling among these participants that the tool generated a lot of useful discussion because it raised awareness of what to consider in using research.

Participants from community-based organizations said that the discussion helped them to understand where the organization was placed with respect to research, because too often one only thinks about one's own immediate environment. This led to the suggestion that future participants could be asked to link the tool to their business or strategic plan, and that this might invoke further discussion. Participants had difficulty differentiating between their own team, department, or the corporation as a whole. There was also some trouble with the apply section of the tool because it was seen as more relevant at the decision-makers level, and participants were not privy to the conversations at this level.

## Discussion

The tool demonstrated good usability and strong response variability in long term care, non-governmental, and community-based organizations. This suggests that the tool is tapping into a set of skills and resources of relevance to research use. Moreover, while the average scores assigned by participants should not be generalized to other organizations in these sectors, the differences between higher-end and lower-end research use organizations on both individual and consensus scores – significant differences for nearly half of the individually scored items and consistently higher scores for 25 of 27 consensus items for higher-end research users – do suggest that this tool has adequate discriminant validity. Time spent on the different sections of the tool varied considerably with the least amount of time and effort expended on the last two sections during the consensus process. Thus, the scores on the latter sections of the tool were arrived at with more limited discussion, and scores may have been modified had more time been available. Our observation from the focus groups was that the more useful aspect of the exercise was the discussion that took place as a result of the item on the tool, rather than the actual score assigned.

The tool was less useful in the government sector, suggesting that additional tailoring of the instrument might be required. Future research might examine whether refinement of the instrument's wording to reflect the government context would render the tool more applicable in this sector.

The breadth of focus groups across sectors, and the number of them, lend to the credibility of findings. Furthermore the approach within each focus group allowed participants to deliberate among them before starting the more formal part of the discussion. This deliberative approach can lead to more informed opinions about issues related to research and how it is used. It also aligns with the learning organization approach, as well as with the creation of collective understanding resulting from the exchange of explicit and tacit knowledge.

The organizational response rate was low. This was due to several factors, including the short time frame available for the study and competing priorities, like an external accreditation process. We believe that the response rate reported here likely underestimates interest in using the tool. Selection bias might have been introduced in the findings as organizations themselves decided who they wanted to invite to the focus group. The mix of participants is likely to have influenced the scores assigned. Although a number of focus groups were conducted, participants and organizations were not selected to be representative of their larger populations. Consequently, it would not be appropriate to suggest that the quantitative findings are generalizable to the four health sectors considered here.

This tool provides a useful starting point for those organizations committed to increasing and/or monitoring their capacity to use research findings to inform decision-making. The study findings have demonstrated the tool's utility in eliciting a provocative group discussion that might generate subsequent action steps or changes within an organization (*e.g*., using a knowledge broker to interpret and implement research in organizations [28]). This reflects the original purpose of the tool and our approach to validity testing. Standard methods to establish psychometric properties were seen as less informative given the way in which users were expected to use the tool in the future.

While organizational team members might complete the tool individually, this initial scoring is a catalyst for a more important group discussion. We observed that the group discussion is, in effect, an intervention. As the data demonstrated, the consensus score did not reflect a simple average of individual scores, but rather reflected a deliberate group process that brought together individual perceptions of research capacity. This discrepancy, and its conceptual meaning, presents an interesting methodological area for future study.

The length of time required to complete the tool suggests that it might be better to complete it during two meetings, when adequate time can be provided for discussion. Anecdotal evidence suggests that many organizations wish to use the tool as a baseline measure of their research capacity, followed by a similar discussion sometime in the future to detect any improvements in research capacity. (We emphasize the point that the tool is meant to explore research capacity rather than performance). Thus, an advantage of a structured tool over simple discussion prompts is the ability to record baseline and post-intervention change in organizational research capacity while maintaining consistent terminology and meanings.

Although we have not examined the properties of the tool related to detecting pre- and post-intervention changes, we offer some recommendations to organizations wishing to move in this direction. Given that the qualitative data from the discussion can yield rich information for the organization to consider, our suggestion is to triangulate the qualitative discussion data with the consensus scores for a more credible interpretation of findings. Further, we suggest that the way in which the initial scoring and group discussion is carried out be carefully documented so that the process can be replicated at the post-intervention time of data collection (that is, consistency in both approach and the people is important to identify change in a reliable way).

Since the completion of this study the foundation has revised the self-assessment tool, incorporating feedback provided by focus group participants in this study. Subsequently, the revised version of the tool the Foundation has received more than 300 requests for this fourth version and is collecting 'lessons learned' and feedback from organizations who have used the tool. Some of these stories are available through the foundation's promising practices series online at .

## Conclusion

Organizations have a role to play in supporting the use of research. While being mindful of the study's response rate, we suggest that the tool presented here can be used to distinguish between organizations that are able to acquire, assess, adapt, and apply research and those that have fewer supports to do so. Further, the distinctions that the tool makes in relation to these four areas are important to identify. The tool can serve as a catalyst for an important discussion about research use; such a discussion, in and of itself, demonstrates potential as an intervention to encourage processes and supports for evidence informed decision-making in the health care system.

## Competing interests

The authors declare that they have no competing interests; MJ became an employee of the Canadian Health Services Research Foundation at the time of manuscript development.

## Authors' contributions

AK participated in the design and analysis of the study, and led the development of the manuscript. NE participated in the design and analysis of the study, and contributed to the manuscript. NH participated in data collection, and helped to draft the manuscript. MJ assisted in the interpretation of findings, and contributed to the manuscript. All authors read and approved the final manuscript.

## Supplementary Material

Additional file 1 **Table 1: Comparison of Individual and Consensus Scores by Higher versus Lower End Organizational Research Users**. Original data used to perform analysis. Click here for file
